# Emerging Trends in Non-Invasive Brain Stimulation: The New Kids on the Block

**DOI:** 10.3390/biomedicines13010014

**Published:** 2024-12-25

**Authors:** Andre R. Brunoni, Paul E. Croarkin, Lais B. Razza

**Affiliations:** 1 Departamento e Instituto de Psiquiatria da Faculdade de Medicina da Universidade de São Paulo, São Paulo 04038-000, Brazil; 2 Department of Psychiatry and Psychology, Mayo Clinic, Rochester, MN 55905, USA; croarkin.paul@mayo.edu; 3 Department of Head and Skin, Faculty of Medicine, Ghent University Hospital, Ghent University, 9000 Ghent, Belgium; lais.razza@ugent.be

In the Sustainable Development Goals of the United Nations for 2030, mental health has been identified as a global priority, emphasizing the need to reduce the prevalence, morbidity, and premature mortality associated with mental disorders. Data from the United Nations highlight that mental health conditions are responsible for 15.6% of years lived with disability (YLDs) and up to 5.1% of disability-adjusted life years (DALYs) across all diseases. Despite these alarming statistics, traditional therapeutic approaches—namely pharmacotherapy and psychotherapy— remain insufficient for many patients [1]. 

In this context, non-invasive brain stimulation (NIBS) has emerged as a promising alternative. Repetitive transcranial magnetic stimulation (rTMS) and transcranial direct-current stimulation (tDCS) are the most established and frequently investigated NIBS modalities. The former uses magnetic fields to induce electrical currents in targeted regions of the brain. This technique, particularly in its high-frequency and intermittent theta burst forms, has shown significant efficacy in treating major depressive disorder (MDD) when applied to the left dorsolateral prefrontal cortex (DLPFC), especially for patients with treatment-resistant depression [2]. While rTMS offers promise, it is not without its limitations. Response rates vary, and some patients do not achieve long-term remission. Conversely, tDCS applies a low-intensity electrical current to modulate neuronal excitability, often used to enhance mood in depressive patients or cognitive function in those with schizophrenia [2]. Despite its ease of use and lower cost compared to rTMS, the clinical efficacy of tDCS is poor [2].

In this Special Issue of *Biomedicine*, studies show that newer NIBS modalities are gaining attention (Figure 1). For instance, temporal interference stimulation (TIS) is a novel type of brain stimulation that uses intersecting high-frequency electrical currents to create a lower-frequency field that targets deep brain regions [3]. This opens up the possibility of modulating areas such as the hippocampus, potentially benefiting patients with neuropsychiatric conditions such as Alzheimer’s disease or major depression, where deeper brain structures play a crucial role in pathology [4]. In this Special Issue, Iszak and collaborators demonstrated that a TIS protocol could modulate, in healthy volunteers, the peripheral nervous system, but not the central nervous system. Remarkably, the authors used neurophysiological surrogate markers such as electroencephalography, phosphene perception, and the direct observation of muscle twitches, observing effects only in the latter readout [3]. The authors further critically discuss the challenges associated with TI. 

This Special Issue also examines another novel form of electricity-based NIBS—magnetic seizure therapy (MST)—a TMS variant that utilizes a magnetic field that encounters no resistance as it passes through the scalp and skull, enabling seizure induction without directly stimulating deeper brain structures. Therefore, this could be an alternative as effective as electroconvulsive therapy (ECT), with less cognitive burden [5]. Here, Bellini and collaborators present the clinical protocol of the EMCODE (Electro-Magnetic Convulsive Therapies for Depression) trial—a randomized, controlled study that aims to compare the efficacy and memory-related side effects of bilateral ECT vs. MST in a sample of 100 treatment-resistant depressed patients [5].

Beyond electrical stimulation, other forms of energy are also being explored. Transcranial photobiomodulation (tPBM), for instance, uses near-infrared light to penetrate the skull and stimulate brain cells; possibly enhancing mitochondrial function [6]. Recent research has shown that tPBM is safe regarding brain heating [7]. However, light penetration through the skull remains a significant challenge, prompting the investigation of alternative delivery paths, such as intranasal PBM [6]. 

Using ultrasound waves, low-frequency transcranial focused ultrasound (tFUS) is a groundbreaking technique that can modulate, with great precision, the neural activity of deep brain structures without the invasiveness of surgical procedures [8]. For instance, translational research has shown that tFUS of the amygdala can modulate fear network activation and connectivity in healthy individuals, which are implicated in several neuropsychiatric disorders [8]. 

As these newer modalities continue to develop, the field of non-invasive brain stimulation is entering an exciting phase. While rTMS and tDCS have paved the way, tFUS, tPBM, TIS, and MST—the “new kids on the block”—represent the next generation of interventions that could offer more targeted, effective, and individualized treatments. Notwithstanding the considerable progress with NIBS, more rigorous clinical trials are needed to realize the full potential of these newer approaches. As we move forward, continued research and clinical trials will be essential to unlocking the full potential of these novel techniques and integrating them into mainstream psychiatric care.

## Figures and Tables

**Figure 1:**
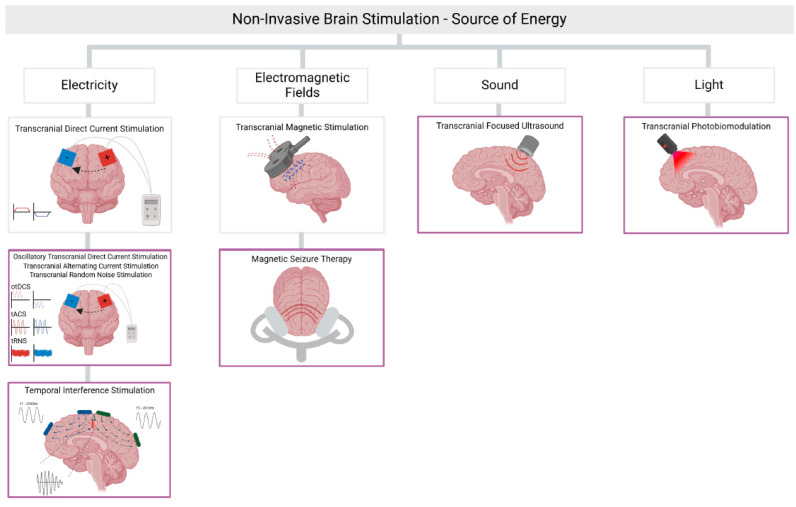
Established and newer forms of non-invasive brain stimulation.
